# The Biosurveillance Analytics Resource Directory (BARD): Facilitating the Use of Epidemiological Models for Infectious Disease Surveillance

**DOI:** 10.1371/journal.pone.0146600

**Published:** 2016-01-28

**Authors:** Kristen J Margevicius, Nicholas Generous, Esteban Abeyta, Ben Althouse, Howard Burkom, Lauren Castro, Ashlynn Daughton, Sara Y. Del Valle, Geoffrey Fairchild, James M. Hyman, Richard Kiang, Andrew P. Morse, Carmen M. Pancerella, Laura Pullum, Arvind Ramanathan, Jeffrey Schlegelmilch, Aaron Scott, Kirsten J Taylor-McCabe, Alessandro Vespignani, Alina Deshpande

**Affiliations:** 1 Analytics, Intelligence and Technology Division, Los Alamos National Laboratory, Los Alamos, New Mexico, United States of America; 2 Santa Fe Institute, Santa Fe, New Mexico, United States of America; 3 Johns Hopkins University-Applied Physics Laboratory, Laurel, Maryland, United States of America; 4 Department of Mathematics, Tulane University, New Orleans, Louisiana, United States of America; 5 National Aeronautics and Space Administration, Greenbelt, Maryland, United States of America; 6 Department of Geography and Planning, School of Environmental Sciences, University of Liverpool, Liverpool, United Kingdom; 7 NIHR Health Protection Research Unit in Emerging and Zoonotic Infections, Liverpool, United Kingdom; 8 Distributed Systems Research, Sandia National Laboratories, Livermore, California, United States of America; 9 Oak Ridge National Laboratory, Oak Ridge, Tennessee, United States of America; 10 National Center for Disaster Preparedness, The Earth Institute—Columbia University, New York, New York, United States of America; 11 USDA APHIS Veterinary Services, Science, Technology, and Analysis Services, Fort Collins, Colorado, United States of America; 12 Laboratory for the Modeling of Biological and Socio-Technical Systems, Northeastern University, Boston, Massachusetts, United States of America; University of Erlangen-Nuremberg, GERMANY

## Abstract

Epidemiological modeling for infectious disease is important for disease management and its routine implementation needs to be facilitated through better description of models in an operational context. A standardized model characterization process that allows selection or making manual comparisons of available models and their results is currently lacking. A key need is a universal framework to facilitate model description and understanding of its features. Los Alamos National Laboratory (LANL) has developed a comprehensive framework that can be used to characterize an infectious disease model in an operational context. The framework was developed through a consensus among a panel of subject matter experts. In this paper, we describe the framework, its application to model characterization, and the development of the Biosurveillance Analytics Resource Directory (BARD; http://brd.bsvgateway.org/brd/), to facilitate the rapid selection of operational models for specific infectious/communicable diseases. We offer this framework and associated database to stakeholders of the infectious disease modeling field as a tool for standardizing model description and facilitating the use of epidemiological models.

## Introduction

While epidemiological modeling for infectious disease is a well-accepted technique for disease management, many epidemiological models do not progress beyond a research study and are not accepted as tools that can be used in an operational setting for infectious disease surveillance. In this context, “operational” refers to the application of an epidemiological model to a real-world event for decision support and can be used as a tool by experts and non-experts alike. The term “model” covers three major types, risk mapping, disease dynamics and anomaly detection. Transition of such models from one-off studies to practical tools is a significant effort that occurs rarely. In addition, even if there are models that could be used in an operational setting, there is neither a standardized approach for describing or assessing models, nor a universal lexicon of terms that allows models to be compared and down selected. To address these challenges, Los Alamos National Laboratory (LANL) has developed a comprehensive framework and lexicon for characterizing epidemiological models that can be used for infectious disease prediction, forecasting and/or monitoring. Such a template could promote an understanding of diverse models by various stakeholders (e.g., model developers, government analysts, public health practitioners, and epidemiologists) with different preconceptions, backgrounds, expertise, and needs, and can foster greater use of epidemiological models as tools in infectious disease surveillance. We describe this framework and our rationale for its development next.

Mathematical and computational models increase our understanding of how different systems function and allow the prediction of their response to modeled changes of concern. In public health, models of infectious disease epidemiology are used for a variety of cases ranging from health promotion and disease prevention to health care system design and operation [1]. When applied to the area of infectious disease management, epidemiological models can be learning and planning tools, and can facilitate public health decision-making. Specifically, models that generate information on how an infectious disease outbreak may either emerge or unfold can provide enhanced situational awareness for decision makers, analysts, and public health officials, and can therefore support prevention and/or mitigation activities. However, instances of epidemiological model use for decision support are few, and adoption of models has been very gradual and various opinions exist on its success [2, 3]. For example, 20 years elapsed before models developed first by Ronald Ross and then George Macdonald were used in the planning for control of malaria [4]. Foot and mouth disease only saw epidemiological modeling used in an operational setting during the 2001 UK outbreak, and discussions of the model’s impact on decision making for mitigating that outbreak have been contentious [5, 6]. The use of epidemiological models is often restricted to experts (model developers) and during an unfolding infectious disease event, it is the experts that discuss findings and relay consensus opinions to policy makers. While this strategy has worked and been praised in a number of independent reports [7], mainstream and routine use of epidemiological models could be implemented if the models could be transformed into operational tools that can be also used by non-experts.

While the intention to apply epidemiological models for various decisions exists, increased implementation faces several challenges. For example, the utility and extent of model application varies greatly depending on the disease of interest and the operational setting. Additionally, actual implementation of the models and policies developed from the model results is not guaranteed [8]. One potential exception to this is influenza because epidemiological models have been used for various aspects of both planning and consequence management of this disease. [9–15].

There exists a plethora of research studies [16] in the literature describing various epidemiological models for infectious disease. However, limited use of these in an operational setting thus far may be due to a lack of accessibility to such models, a limited availability of web-accessible models, and a lack of plug and play types of models. In addition, there are two other specific challenges to integrating epidemiological models into operational biosurveillance that are rarely considered:

### 1. Determining the operational context of a model

Choosing a useful epidemiological model for a given outbreak type is impossible without knowledge of context and level of pre-operational development and testing [16], that is, without a geographic or population scale of application and/or an understanding of available data and other information sources, it is very difficult to determine what epidemiological model is appropriate to use or whether a particular model is readily available for use. Moreover, beyond having a graphical user interface (GUI) or web accessible interface, the components that make a model “operational” are yet to be well defined. Such components must resolve basic questions like:

- What is the purpose of the model? What decisions is the model designed to support?
- What surveillance goal does the model address?
- Is the model currently in use? If so, how, where, and by whom?
- Has the model been verified and validated?
- For which regions of the world does the model apply?
- What data inputs and data streams are required to generate a prediction or forecast?
- What computational infrastructure and resources are required to run the model?
- Can the model’s capability be extended to include a new disease and/or location?

These are all relevant questions that are infrequently answered in the literature. Without answers to these questions, the selection of a context-appropriate model is impossible.

### 2. A lack of consistent model characterization schema that allows a user to make an appropriate manual comparison of available models

Given the diversity of model types and the manner in which they are described, the selection of an appropriate model is also a challenge. As an illustration of this challenge, consider the websites for the North American Animal Disease Spread Model [17] and EpiMAN-FMD [18]. Both are generic “operational” platforms focusing on livestock disease. However, the two platforms are described very differently even though the underlying model structure is similar. A non-expert in modeling would find it difficult to make a choice between these platforms primarily because they are not described in a common framework using a common lexicon. Underlying techniques and uses of models are often interchangeably used to describe models. For example, “risk mapping” and “hot spot mapping” are often used interchangeably to identify a model type, though risk mapping is a type of model [16] while hot spot mapping is a technique applied to identify a risk area.

There are ongoing efforts by public health stakeholders in government, academia, and industry to increase the use of epidemiological models in infectious disease management. For example, to increase use of predictive modeling of epidemic emergence for public health preparedness, requirements for effective, sustainable models are provided by the Centers for Disease Control’s (CDC) Framework for Preventing Infectious Diseases [19]. The National Operational Epidemiological Modeling Process (NOEMP) is a set of guidelines proposed by Lenart et al. [2] to support improved information management in a health emergency or disease outbreak. One of the proposed functions of NOEMP is that this framework “enhances the use of models during an operational response, including sharing models and model outputs within and between agencies/organizations”. While these efforts are a first step towards bringing epidemiological models into mainstream operational surveillance of disease, standards are required to facilitate their use and acceptance. For instance, the definition of “operational” itself can depend on who the targeted users of models are, the specific use, and the technical skills of the users. Thus, a key need is a universal framework to facilitate model description and an understanding of its features; development of such a framework and the associated database is the primary goal of the work presented in this paper.

LANL developed a comprehensive framework to facilitate characterization of infectious disease models in an operational context and facilitate manual model comparison. The framework was developed through a consensus among a panel of subject matter experts that included both model developers, model users and decision makers. In this paper, we describe the framework, its application to model characterization and the development of a web-based directory, the Biosurveillance Analytics Resource Directory (BARD), to facilitate the rapid selection of operational models for specific infectious/communicable diseases. We offer this framework and database to the stakeholders of the infectious disease modeling field as a tool for standardizing model description and thus enhance the use and development of infectious disease models in operational settings. One of the objectives of the model characterization framework is to encourage model developers to start thinking and describing the many features of their models using a common format. We illustrate the application of the framework through the development of the BARD which is a scientific and non-biased tool for selecting an appropriate epidemiological model for infectious disease surveillance.

## Materials and Methods

To develop a conceptual framework for characterizing models used for operational disease prediction, forecasting, and monitoring we collected information via a three-step process: a literature search of modeling characteristics, a review of current operational infectious disease epidemiological models, and subject matter expert (SME) panel consultation.

A literature search was conducted to inform us about terms used by infectious disease model developers and researchers to characterize their models. The purpose of the literature search was not to provide a comprehensive review of modeling terms, but rather to understand modeling terms, how they were used in the field, and the discrepancies or redundancies in meaning found in the term’s usage. Multiple sources from a variety of modeling fields were consulted including ones outside of infectious disease modeling like book chapters, conference proceedings, peer-reviewed literature, reports found through traditional database searching (e.g., Web of Knowledge, Google Scholar), as well as textbooks and model websites. The search was intended to seek out the broad spectrum of vocabulary used in published literature related to infectious disease modeling or to modeling approaches in general. Definitions, descriptions, and operational context terms were tabulated and cross-referenced to develop common terminology and were collated into a preliminary framework to describe and compare models.

A process similar to our literature search was followed to identify relevant infectious disease models and to evaluate the effectiveness of the framework in characterizing models. We limited the initial selection of models to five infectious diseases: influenza, malaria, dengue, cholera and foot-and-mouth disease (FMD). These diseases capture a variety of transmission modes (human-to-human, vector-mediated, environmental exposure), represent high or potentially high epidemic or endemic burden, and are well represented in the literature. Models were identified through web searches, literature searches (similar to above), and information provided by our SME panel. Our team also developed working criteria (Table 1) for what attributes can be used to comprehensively describe an operational model. These factors, described in Table 1, include consideration of a model’s documentation, accessibility, and sustainability and are listed in decreasing order of maturity.

**Table 1:** Working Criteria for “Operational” Models.

Model Documentation	Model Association	Model Distribution
Model has the equivalent to a ‘user manual’	Model is currently in use and is being actively promoted for use by others	Model code/software is readily available either by open-source, by subscription/registration, or by purchase
Model has technical documentation	Model is linked to a website specific to the model	Model code/software is available for distribution for limited use
Model has been only described in peer-reviewed literature	Model is only linked to a research group through documentation (such as research team from a university that has published an article describing results)	Model code/software is not accessible/available

A subject matter expert panel was assembled that included model developers, decision makers, and analysts with expertise and/or experience in the use or development of epidemiological models for decision-making. Experts were selected by our team on the basis of their demonstrated expertise (publications or technical presentations) within the subject matter of the use or development of epidemiological models for decision-making, or on the recommendation of an expert. The panelists consisted mostly of experts from the United States who work in academia or government; however, there were a few experts from other nations and industry. Table 2 includes information regarding our SME panel.

**Table 2:** SME Representation.

**Total attended demonstrations:**	29 SMEs
**Affiliation Representation:**	
Academia (10)	John’s Hopkins University Bloomberg School of Public Health, John’s Hopkins University Applied Physics Laboratory, University of Kansas Lawrence, Northeastern University, The Santa Fe Institute, Tulane, University of California Davis, University of Liverpool, University of Maryland, Virginia Tech Virginia Bioinformatics Institute, Yale
Industry (1)	IBM
Government Agency (7)	Centers for Disease Control, Department of Homeland Security (DHS), DHS/National Biosurveillance Integration Center, Defense Threat Reduction Agency, DHS-Foreign Animal Zoonotic Diseases, National Aeronautics and Space Agency, National Surveillance Unit, United States Department of Agriculture (USDA)
National Laboratory (4)	Los Alamos National Laboratory, Sandia National Laboratory, Oak Ridge National Laboratory, Pacific Northwest National Laboratory

The SME panel had three purposes: 1) to provide information on modeling terms and their usage, 2) to provide the names and information of relevant infectious disease epidemiological models, and 3) to provide feedback on the model characterization framework and the resultant tool, the BARD. The panelists were provided with a document that described the modeling terms and definitions, and model characterization framework as well as questions regarding the functionality and usability of the BARD. Additionally, each panelist attended a teleconference and video demonstration of the BARD. The panelists provided oral and written feedback on the BARD and the characterization framework. Based on SME feedback the terms and definitions, characterization framework, and database tool were refined. The updated characterization framework was provided to the SMEs for a second written evaluation.

Formalization of model characterization framework, terms and definitions–It is important to note that this project is a first effort towards making model descriptions standardized, and terms and definitions for the framework were derived through numerous sources described in the paragraphs above, and vetted with the SME panel. This was an iterative process that took several months and the framework and glossary (S1 File) underwent refinement following each teleconference, e.mail notification and video demonstration. The final products shown in this paper are consensus definitions which cannot be seen as “formalized” until official entities such as MIDAS (Models of Infectious Disease Agent Study; http://www.nigms.nih.gov/Research/SpecificAreas/MIDAS/Pages/default.aspx) are willing to adopt and promote their usage. This paper is a means of starting this dialogue and spreading the word in the global epidemiological modeling community.

Only contact and affiliation information was collected about the individual SME responding to the questionnaire, and the survey was strictly a means to record expert opinion. Therefore, the SME panel survey did not involve human subjects research, and institutional review of the survey was deemed unnecessary (Common Rule (45 CFR 46), LANL Human Subjects Research Review Board (HSRRB)).

**Fig 1:**
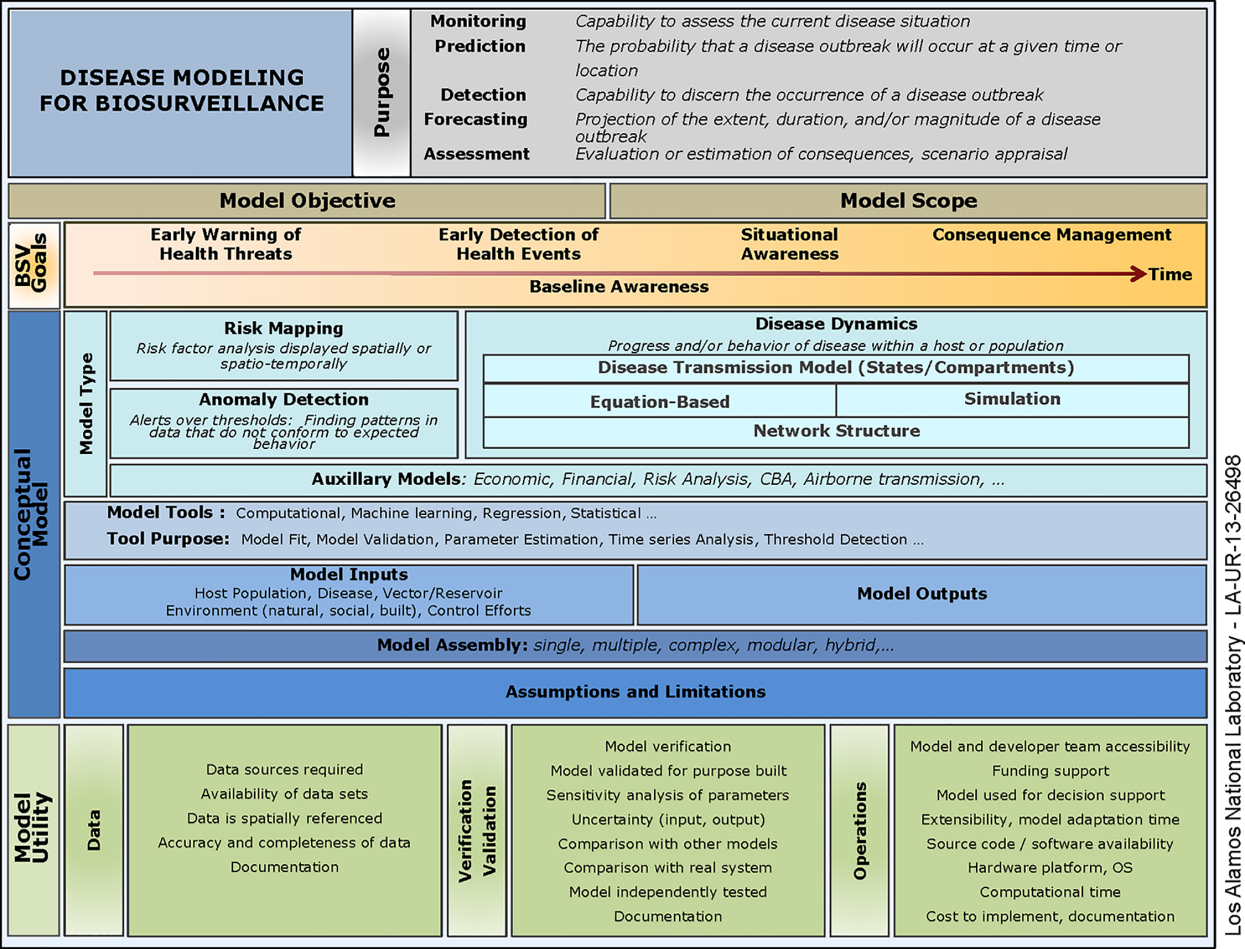
Model characterization framework that includes 6 major components.

To apply the model characterization framework, we built a searchable relational database. This database, the BARD, is currently a prototype but is publicly available (http://brd.bsvgateway.org/brd/). The database is not necessary to replicate the findings from our study, but is meant to demonstrate the utility of our framework. BARD is meant to be a searchable reference of epidemiological models. The models, modeling terms, and systems described are current as of June 2015. Descriptions of terms exist in words, but have not yet been formalised as computer readable terms. Many of the terms could be linked to terms in existing ontologies such as those available in Bioportal (http://bioportal.bioontology.org/) thus allowing the linkage of multiple resources. This can be an added advantage for epidemiological models included in an open repository such as the BARD. Categories in the BARD are based on the model characterization framework and aim to capture all relevant information about epidemiological models. The database schema is based on our model characterization framework that has been explicitly described in Supplement 1 (S1 File; Model glossary) and shown in Fig 1 (Fig 1). The schema includes categories, and associated terms which are also defined in the Model Glossary. A document was also developed to describe the usability requirements for the BARD; that is, potential users (and non-users) are formally described in order to elucidate how the BARD will be utilized (S2 File). The document allows a reader to understand the specific types of users that will benefit from the use of the BARD and the functions/tasks it will facilitate. It does not include information on technical implementation (e.g., whether specific information is contained in the database or pulled on demand from other sources). It also avoids specific design ideas (such as widget descriptions) unless they are necessary to illustrate a specific requirement.

To characterize the models using the framework, we did a literature search for papers and technical documentation. We also searched for websites that host the models. Two separate members of our team went through documentation of the model and assigned properties to each of them. Properties for which the assignments agreed were entered into the BARD. The model developers were then contacted and asked to fill in properties for fields for which the assignments did not agree or the documentation was not sufficiently detailed. Model developers were also asked to verify the accuracy of the information included in the BARD for their models.

## Results

### Framework for model characterization

LANL developed a framework for systematically characterizing epidemiological models applied to infectious disease surveillance that could be used for selecting such models in an operational setting as well as making manual comparisons between similar models at a high level. The framework and the BARD can be used by model developers to both describe their models and offer them to decision makers for operational use. The BARD can also be used by analysts and decision makers to rapidly select models during the planning or response of a real world biological event. The framework was scoped by the core functions outlined in the National Strategy for Biosurveillance [20] with the intention to facilitate better decision-making. A more detailed description of the biosurveillance context (goals and data sources) can be found in Margevicius et al. [21]. The *modeling framework* (Fig 1) enables models to be characterized not only by the mathematical constructs underpinning their application but also by scope and operational utility.

The framework is divided into six major components: Model Purpose, Model Objective, Model Scope, Biosurveillance (BSV) goals, Conceptual Model and Model Utility; each of which has several sub-categories for characterizing each aspect of a model. For each of these components, a list of terms and definitions has been compiled, the full detail of which is available in S1 File.

**1. Model Purpose** describes the specific function of the model and the question it is addressing; e.g. disease prediction or forecasting, assessment, monitoring or detection. While “prediction” and “forecasting” have been used interchangeably as a description of the type of model being used within a paper, we define prediction as the probability that a disease outbreak will occur at a given time or location and forecasting as the projection of the extent, duration, and/or magnitude of an ongoing disease outbreak ([16] S1 File).

**2. Model Objective** describes the specific reason for developing and implementing the model such as planning for a vaccination strategy or predicting case incidence.

3. **Model Scope** identifies the specific location or disease for which the model has been designed and whether the model is a general platform that is configurable for different diseases and locations.

**4. BSV Goals** refer to the specific biosurveillance goals developed by LANL that cover the entire time spectrum of biosurveillance from pre-event warning and detection to consequence management [20]. This component, together with Model Utility (described as component 6 below)**,** allows for a model to be considered in the context for which it has been developed and for which it might be used.

**5. Conceptual Model** characterizes how a model predicts, forecasts, or monitors and describes aspects such as the mathematical framework used, the model inputs and outputs and the modeling techniques. Disparate types of models that are variously described in the literature can be easily distinguished; e.g., risk mapping, anomaly detection, and equation- and simulation-based disease dynamics models. By using the term Conceptual Model, the overall approach of the model can be described and separated from the details associated with the operational aspects of the model. This component also includes sub-categories to capture specific information on tools used by the model, the purpose of the tools, inputs and outputs of the model, and assumptions and limitations of the model (S1 File). Categories like assumptions and limitations for a model can have very diverse content, and rather than providing a drop down list with fixed vocabulary, we allow free form text entry as is illustrated in Table 3. Essentially, these fields are a means of offering the user as much information on the models so that they can make an informed decision. It may not be possible to do an “apples to apples” comparison using one or more singular categories, but the hope is that the overall description using this framework provides a first step in that direction.

**Table 3:** Framework Use Cases.

	Risk Mapping	Anomaly Detection	Disease Dynamics
	Liverpool Malaria Model 2010	SaTScan	EpiSimdemics
		
**Purpose**	Forecasting	Detection	Assessment (Situation Assessment, Planning and counterfactual analysis)
**Objective**	Predict expected disease cases	Inform disease process—Identify disease cluster	Policy planning and course of action analysis
**Scope**	Specific disease Application	Platform	Platform
**BSV Goals**	Early warning (EW), Early detection (ED)	ED, Situational Awareness (SA)	SA, Consequence Management (CM)
**Conceptual Model**			
Model Type	Disease dynamics	Anomaly detection, Poisson based; Bernoulli model	Disease dynamics
Model Tools /Purpose of tools	Epidemic, SEIR model/ movement between disease states	Statistical/ Determination of significant clusters in space or in space and time (Threshold detection)	Epidemic, SEIR model/movement between disease states
Model Inputs	Disease, Epidemiological; Vector; Environmental, Climate	Disease, Epidemiological; Environment, Geography; Host Population, Demographics	Control Efforts; Disease, Epidemiological
Model Outputs	Disease incidence, Epidemic spread	Disease incidence	Control effort effectiveness, Disease incidence, Epidemic spread
Model Structure	Compartmental model, developmental degree days	N/A	A general finite-state machine, or probabilistic, timed transition system
Assumptions	Uses literature based parameter settings	Number of events in a geographical area is Poisson-distributed	Not clearly delineated
Limitations	Broad scale no local land use conditions		The class of diseases; cannot be applied to vector disease epidemiology
**Model Utility**			
Data			
Data Sources Required	Daily temperature, Rainfall, vector life cycle	Depends on specific application (can be clinic/health provider records, lab records, ED/hospital records, established databases)	Established databases (US census, NAVTEQ street data, databases for non-housing locations, school, and activity data)
Data Availability	Good from modeled data sets i.e., reanalysis or forecast/climate model archives		Good
Documentation	Yes	Yes	Yes
V&V			
Verification	Yes	Yes	Yes
Validation	Yes, Using a clinical diagnosis based malaria index 20 years + from Botswana	Validated with Ground-truth data	Used by Federal agencies for pandemic planning
Sensitivity Analysis	Yes	Unknown	Yes
Uncertainty	Normally run with multiple driving data (climate) sets to encompass some of the uncertainty.	Uncertainty clearly documented	Not documented
Documentation	Yes	Yes	Yes
Operations			
Developer Team Accessibility	Model team still active	Unknown	Model team still active
Funding	Currently funded	Currently funded	Currently funded
Extensibility	By disease and location	By disease and location	By disease and location
Source code / hardware availability	No, but desktop version available	No	Yes upon request
Computational requirements	Low (desktop)	Low (desktop)	High (super-computer)
Cost to implement	Software is free	Software is Free	Unknown
Documentation	A tutorial and training data set is included in the desktop version	User manual available	Not available

Often, a disease model is used along with other models that evaluate risk or perform cost-benefit analyses to achieve a particular objective (e.g., ensemble models that link risk mapping or disease dynamics with economic models). While the specifics of these “hybrid” model types are not completely characterized, the Conceptual Model framework component articulates the use of each model type in a hybrid. This component also explicitly categorizes model tools, inputs, outputs, assumptions and limitations such that through precision of terminology, commonality or differences in these features among can be identified.

**6. Model Utility** describes various operational features of a model such as the type of data it may use, whether it has been validated and verified, the level of readiness for decision support (e.g. run time, accessibility, hardware platform, computational time, etc.). This component of the framework was created to help determine whether a model may be rescaled or otherwise modified for a context (e.g., disease type, location, output product) beyond that for which it was designed. This component also assesses required development stages for modification and implementation; i.e., Data, Verification and Validation, and Operations. The usefulness of a model in decision making may rely heavily on factors such as data availability and the availability of the model design team to assist in assuring appropriate and relevant model use. Having the model utility information characterized alongside the more technical aspects of a model supports decision making both in research and in operational settings.

### Application to model characterization

Models for five infectious diseases—cholera, malaria, influenza, FMD and dengue were characterized using the framework and are included in the BARD database. A total of 53 models were characterized and include epidemiological models that predict, forecast or monitor these diseases. The models were selected using the criteria described in the Methods section. The characterization of models illustrating the three primary model types in our framework (risk mapping, anomaly detection and disease dynamics) is shown in Table 3. Three models—the Liverpool Malaria Model] LMM, [22], SaTScan [23], and EpiSimdemics [24] were selected as examples to illustrate our characterization framework in this paper. Availability of detailed information for these models in the reviewed literature as well as the ability to easily contact the model developers were criteria used for this selection.

Our framework characterizes disparate models in a streamlined fashion as all model information may be binned into the same categories, allowing easy manual comparison and understanding of the models. For the three models, four of the six components (Purpose, Objective, Scope, and BSV Goals) were fairly straightforward to describe. However, Conceptual Model and Model Utility components had to be further divided into several sub-categories to best capture finer details as shown in Table 3 and S1 File. In the Model Utility component these include Model Type, Tools, Inputs, Outputs, while the Model Utility component sub categories include Data Sources, Documentation, Verification and Validation. Within these numerous sub categories, we aimed to stay at a higher level of information description to facilitate both information understanding as well as input. For example, under the “Model Inputs” sub-category, for the model EpiSimdemics, description was limited to “control efforts” rather than a detailed list of exactly what kinds of control efforts specifically used. Similarly, a model input “demographics” for SaTScan is sufficient to inform the reader without the need for specific details on the various types of demographics used. However, details for each sub-category are made available in the BARD.

Specific definitions for each category used in the framework also facilitated input of appropriate model information. For example the term “Verification” is defined in our Glossary as “conceptual model has been adequately translated into formula or computer code and performs as intended—no coding or logic errors”. Validation, on the other hand, can occur through different means (comparison with other models, comparison of model with real system, model tested outside of developer team), any of which would result in a “yes” to address that sub-category of model information.

Certain fields required in our framework were often omitted within model documentation. In particular, the sub categories of model limitations and assumptions (under the Conceptual Model component) were often not explicitly described or discussed in adequate detail. Additionally, many operationally-relevant details such as the source code language and availability, computational and operating system requirements, verification and validation (V&V), and the time it may take for a model to be adapted to a different disease or location were often not discussed. While this aspect is understandable since much of the documentation were academic papers, for models to be used in operational settings, these types of details need to be included in the model characterization/categorization.

### Development of the BARD

The BARD is a decision support tool that can be used to search for and rapidly select appropriate, epidemiological models that may predict, forecast or monitor infectious diseases. Rather than being a conceptual classification scheme, our model characterization framework is implemented in this actionable tool currently in prototype stage. The BARD provides specific information about a model that has been systematically categorized and will offer precision through its search and find function that is not available if one were to perform a standard Internet search. The BARD allows manual category-to-category comparison of multiple models in support of a single disease and provides links to specific models with updated and accurate contact information for each model facilitating its immediate use. The tool can also be used to characterize new models that may be included in the future. The BARD was built as a resource to efficiently obtain and retain specific information about each model, as characterized by the model framework. It is important to note that the *BARD is not a tool to rank models* but rather a source of model information that is easily available to a user in a format that allows the *user* to make a ranking or an assessment of the utility of the model. It does not evaluate or verify the accuracy of the models as that is a much larger effort and beyond the scope of the current project. Thus, the BARD does not guarantee the accuracy of the models included in it, but does provide information about whether the particular model has been verified and validated by the developer or an independent entity.

To facilitate a first pass in finding models that could be considered useful in an operational setting for each of the five specific diseases and included in the BARD, we sought models that met the criteria described in the Methods section. Note that for the five diseases being included in the prototype BARD, only a small percentage of models among those for which information was collected met these criteria for being operationally mature (Table 4). *These constitute models that have been transformed into tools for decision support*. A second tier of models was included into the BARD if they had unique model purpose or technique but were not necessarily mature as tools.

**Table 4:** Models included in the BARD.

Number of Models	Influenza	Cholera	Dengue	FMD	Malaria
**Described Research Papers**	109	30	22	81	34
**Included in BARD**	13	11	10	15	4

The results of our initial compilation and characterization of models for cholera, dengue, FMD, influenza, and malaria in the BARD shows the usefulness of the characterization framework in providing relevant information to an analyst or practitioner in selecting models in an operational situation. The results may reflect the bias of our team's discovery efforts, and determination of which models to include in the BARD. However, a next stage in the development of the BARD is to include a web-based, user interface for the BARD that would allow users of the BARD to create, edit and provide feedback on content and functionality as well as to maintain curated information in this database through a community driven effort.

**Fig 2:**
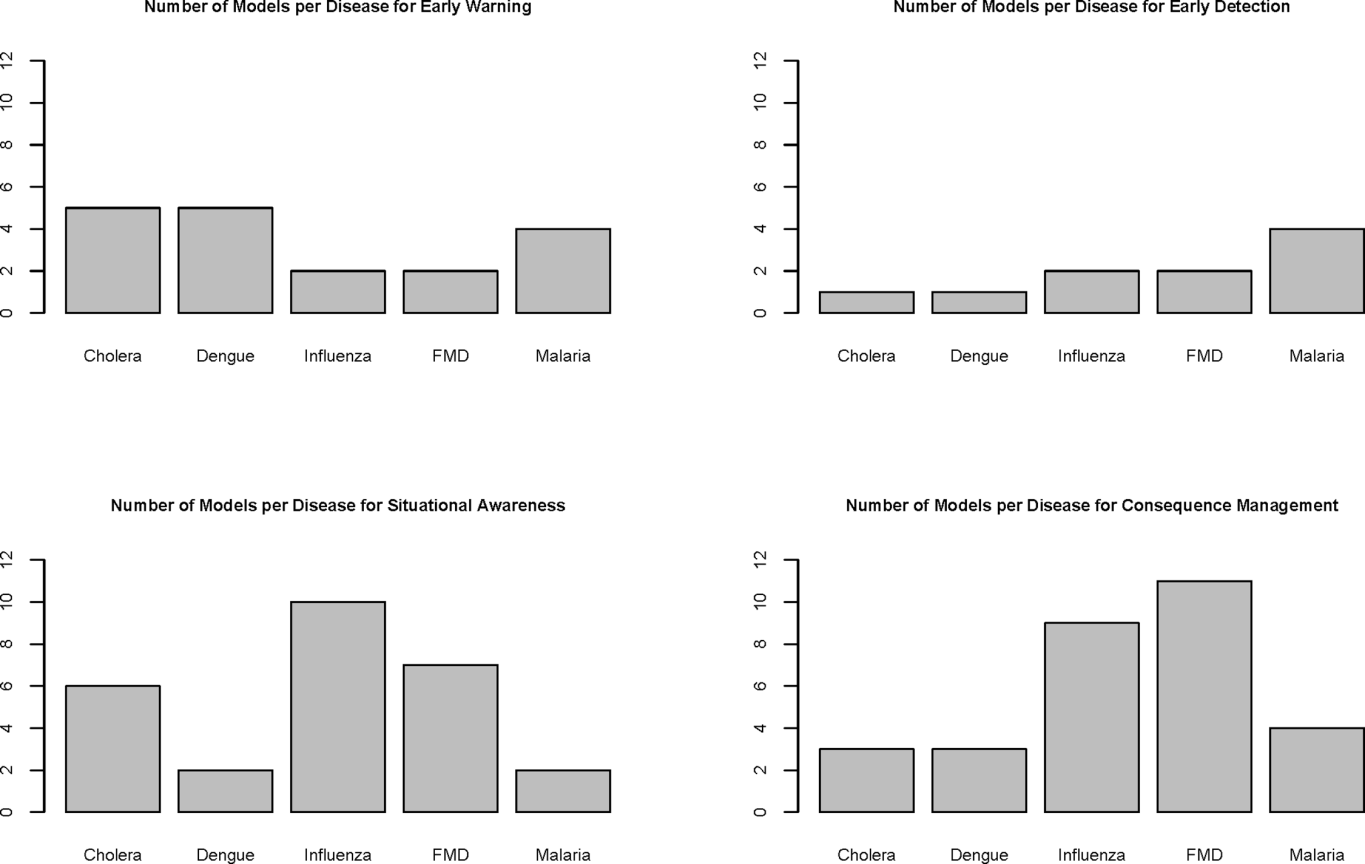
BARD models developed for a particular biosurveillance goal for a specific disease

Fig 2 shows the distribution of models in the BARD developed for a particular biosurveillance goal (early warning, early detection, situational awareness, consequence management) for a specific disease. Other than models for dengue, and perhaps cholera, more models are available further along the timeline of biosurveillance (i.e., for situational awareness and consequence management) than for early warning or early detection. Such a form of searching reveals surveillance goals for which model development could be directed.

**Fig 3:**
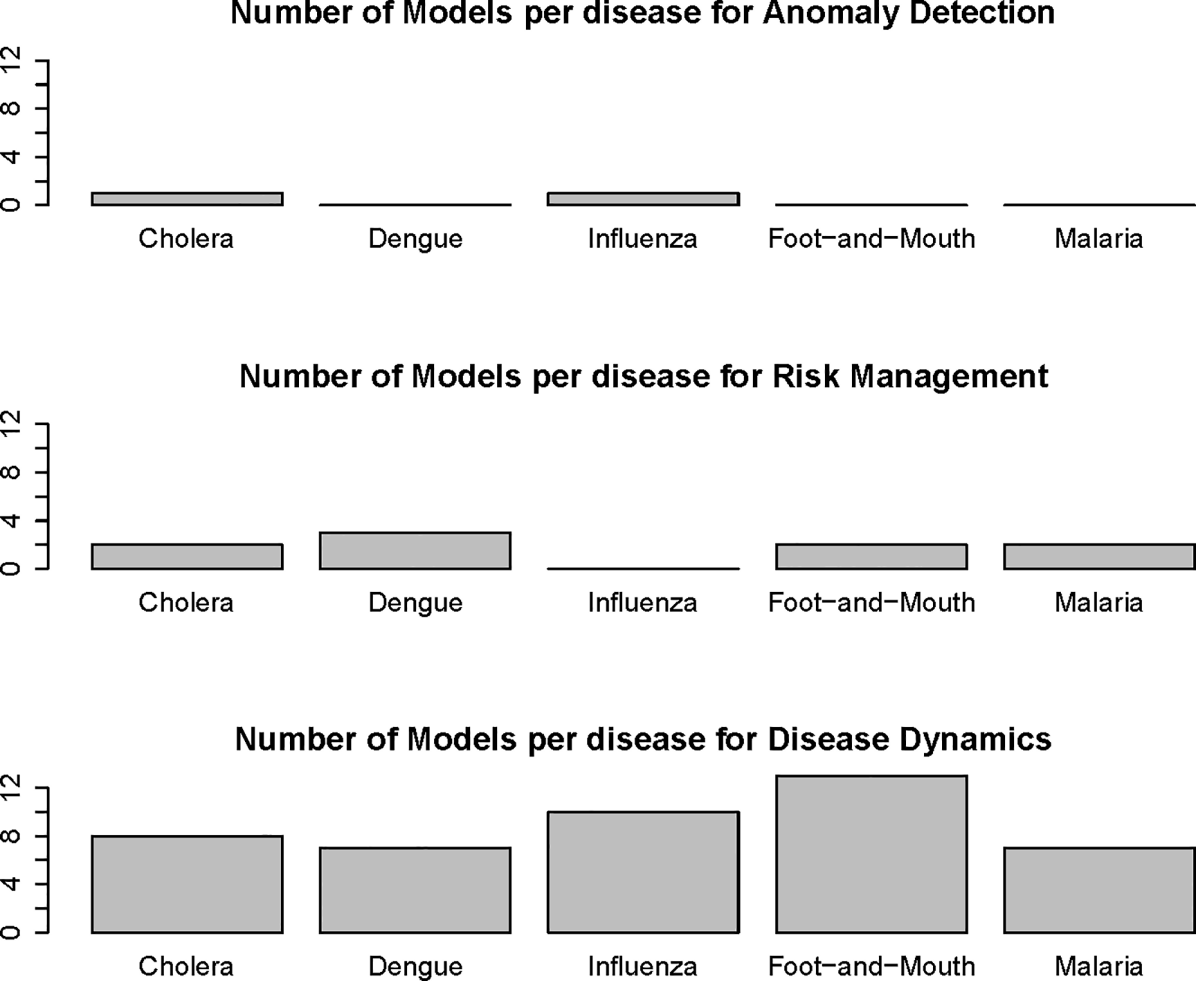
Types of models in the BARD

For all diseases, the most represented model type (risk mapping, anomaly detection, disease dynamics) was a dynamical model of disease transmission (Fig 3). This holds true even when looking at only those models that have been transformed into decision support tools. Anomaly detection models are often closely linked to disease surveillance systems and are not necessarily called out as separate models. For example ESSENCE is an anomaly detection tool that is already operational and associated with several US state surveillance systems (e.g., ESSENCE-Florida, [25]). This may be the reason why anomaly detection models were not well represented.

## Discussion

Mathematical modeling and simulation are potentially powerful tools that are becoming increasingly important for public health decision-making and disease prevention strategy and policy. However, models are not necessarily being developed with decision makers in mind. This gap between model developers and decision makers needs to be narrowed before modeling becomes routinely used to inform decision makers during biological events. The gap is reflected by the difference in the modeling numerous models and modeling techniques published each year and the small number of these which have been used operationally. Of those few that have been deployed in operational capacities, an even smaller number are described in enough detail and with specific guidelines describing their operational characteristics, verification and validation information, technical documentation, readiness and utility. To further encourage model use in rapid decision making, they must be transformed into tools that can be also used by non-experts in the field of modeling and simulation.

The persistent gap between developed and operationally ready models is not the fault of any single party, but rather is a consequence of a complex situation involving multiple types of institutions and jurisdictional levels with disparate goals amid a changing infectious disease environment, with goals swinging between bioterrorism prevention and the routine needs of public health surveillance. Front line health monitors have had little influence on the selection and development of analytical methods. Model developers are not provided with guidance (or funding) that addresses what under-resourced front line public health practitioners operationally need from their model as well as characterization of their diverse and often ill-defined situational awareness needs. Moreover, Academia itself doesn’t reward developing operational models, it rewards publishing papers. Funding for advanced analytic and surveillance capabilities, especially at the local level where surveillance is often practiced, is ephemeral when available at all. More often than not, local government public health organizations struggle to find the funding to meet basic services in their jurisdictions, let alone develop advanced analytical and surveillance capability.

The characterization framework and the resultant tool developed—the BARD—represent a first step towards working to solve these problems. They offer the initial steps of an infectious disease epidemiological model ontology that can be understood and used by stakeholders who may not have a modeling background. Our tool will be a significant step in bringing many models out of the purely academic realm and into an operational setting as it describes models in a standardized and structured fashion, with a common lexicon of terms. There are several efforts underway in different communities to do this (e.g., biological process models, ecology, [26–29]) and a similar initiative is required for epidemiological models used for infectious disease surveillance.

Key features of these efforts have been the development of standardized model repositories such as the Biomodels database maintained by the European Bioinformatics Institute, and the development of a centralized repository of relevant biological ontologies (http://obofoundry.org) that can be referenced in the biological community. Together these initiatives contribute to a standardized process by which biological pathways and processes are modelled, described and understood. Systems biology standards are commonly developed under the umbrella of the Computational Network for modeling in Biology, COMBINE (http://co.mbine.org).

The Biomodels database houses models developed for biological processes and pathways that can be downloaded by users (https://www.ebi.ac.uk/biomodels-main/). There are two main differences between the BARD and the Biomodels database;

1) The BARD only serves as a repository of information about epidemiological models that have been described using a common lexicon of terms. It does not house the models per se, but rather links to websites and contacts that can be used to obtain the models.

2) Consequently, there is no verification and validation of models but rather a display of relevant information that allows a user to make an informed decision about the appropriateness of a model. This is primarily due to the current scope described for the BARD. That is not to say that this repository of information could not be modeled along the lines of the Biomodels database and eventually become similar to it. Additionally, we are working towards interlinking models and metadata within the database and continue to add this functionality as we refine the content for each model. Such features are already implemented in the BioModels Database.

Epidemiological models are far more diverse than biological models in terms of not only their scope and range of application, but also their mathematical underpinnings and operational characteristics like the language they are written in, the platforms they use, the computational power they require and the data inputs used. This makes building central storehouse of models all the more difficult and will likely have numerous challenges. However, describing models and building a repository of model information is a first step towards encouraging the disease surveillance community towards a way of thinking adopted by members of the biological modelling community. This database is the first available system that collects models.

Existing models can range from highly sophisticated data-driven approaches that require expert training to very simplified models with user-friendly GUIs and no complex computational requirement. The uniformity that the characterization framework and the BARD offers for assessing and comparing models gives a wide variety of stakeholders clearer choices and means to implement models relevant to their situation without having to undertake a lengthy period of searching via published outputs and websites (when they exist) of these models. For example, consider a scenario wherein an analyst would like to make projections of how a malaria outbreak may unfold. For such a scenario, specific information about an operational model categorized using standardized vocabulary, in a searchable database is more useful than weeding through thousands of hits obtained by a generic web search for “operational models for malaria”. In addition, the analyst might be able to apply a usable model and save time during an unfolding event rather than having to go through the process of getting together SMEs to inform a decision that may be needed to quickly mitigate the event.

The BARD offers a way for decision makers to make informed model selections because it provides information on epidemic modeling features included in the model and the overall usability of the model in terms of input requirements and computational resources needed in one location. Additionally, the database allows the user to understand the limitations of each model and what is required to drive the model. It gives a clear indication of the type of outputs and thus decision making questions that can be undertaken by each model. A further advance in this tool could be the use open source ontologies and semantic annotation to refine the model characterization frameworks and add more detail to model description to enable better manual model comparison. For example, the epidemiology ontology offered by the Open Biological and Biomedical Ontologies (OBO) foundry (http://www.obofoundry.org/cgi-bin/detail.cgi?id=EPO). While not directly related with an epidemiological model, such a resource could help refine the metadata descriptions of a model such as purpose and scope.

A consensus-driven approach together with the long-term sustainability of a tool like this does have limitations and challenges. Maintaining stakeholder consensus and communication is a difficult, but necessary, process in order for this approach to be effective. Additionally, there are many challenges to practically operating and sustaining a tool like the BARD over time. To maintain its relevance, BARD content needs to be updated on a regular basis and as a result, there needs to be some level of continual financial support. One possible way to ameliorate the difficulty of these challenges is by operating the BARD and the characterization framework as a community-driven tool whereby the community can update the BARD in much the same way as a wiki. By allowing users to submit feedback as well as to offer changes or edits to entries (that would need to be approved by community-chosen editors), costs associated with maintenance could be significantly lowered while engaging stockholders at the same time. This opens up many exciting possibilities for community engagement. For example, the usefulness, efficacy and robustness of a model can only be determined through extensive testing beyond the research team that developed the model for its own project(s). Therefore, it will useful to include a user feedback section in the BARD. If the model has already been distributed either openly or on a limited basis to the infectious disease or public health communities, the BARD’s responsible official can solicit and moderate comments from the user communities.

With a growing need for mathematical modeling, the effort necessary to evaluate and select a suitable model for a particular issue can be daunting. The BARD provides a resource to minimize the time and effort, as well as to expand the search for existing models. The tool allows decision makers to plan and pre-position models so that they are prepared in the event of needing to use a model for operational response. Prior to or in the face of an outbreak, this can be invaluable. There have been recent efforts to produce models that can be used by decision makers not necessarily having expertise in modeling, through the addition of easily accessible user interfaces and visual aids (e.g., the suite of web-based apps developed by Virginia Bioinformatics Institute, [30]; FRED, [31]; GLEAM, [32]; the Texas Pandemic Flu Toolkit, [33]). However, without a standardized description of the underlying model and its operational use, there exists the potential for misuse and misinterpretation of results as well as a lack of confidence in them [34]. LANL’s model characterization framework and associated web-based model database offer the first steps towards increasing the acceptance and use of epidemiological models for infectious disease prediction, forecasting, and assessment.

## Supporting Information

S1 File Glossary of model framework terms and definitions. (PDF)

S2 File BARD usability document. (PDF)
