# Supplementary material for: The Biosurveillance Analytics Resource Directory (BARD): Facilitating the Use of Epidemiological Models for Infectious Disease Surveillance
Source: PLoS One. 2016 Jan 28;11(1):e0146600. doi: 10.1371/journal.pone.0146600 (PMC4731202; doi:10.1371/journal.pone.0146600)
Supplement: S1 File — (PDF) [file pone.0146600.s001.pdf]

Supplement 1 – Model glossary

Towards a standardized framework for describing epidemiological models: characterizing models in an operational context

Kristen J Margevicius[1], Nicholas Generous[1], Esteban Abeyta[1], Ben Althouse[2], Howard Burkom[3], Lauren Castro[1], Ashlynn Daughton[1], Sara Y. Del Valle[1], Geoffrey Fairchild[1], James M. Hyman[4], Richard Kiang[5] , Andrew P. Morse[6], Carmen M. Pancerella[7], Reid Priedhorsky[1], Laura Pullum[8], Arvind Ramanathan[8], Jeffrey Schlegelmilch[9], Aaron Scott[10], Kirsten J Taylor-McCabe[1], Alessandro Vespignani[11] and Alina Deshpande[1*]

Model: Framework

Model Purpose, Objective, Scope

Model Purpose

Monitoring
Capability to assess the current disease situation

Prediction
The probability that a disease outbreak will occur at a given time or location

Detection
Capability to discern the occurrence of a disease outbreak

Forecasting
Projection of the extent, duration, and/or magnitude of a disease outbreak

Assessment
Evaluation or estimation of consequences, scenario appraisal

Understood in the context of biosurveillance:

"The process of gathering, integrating, interpreting, and communication essential information related to all-hazards threats or disease activity affecting human, animal or plant health, to achieve early detection and warning, contribute to overall situational awareness of the health aspects of an incident and to enable better decision making at all levels" - National Strategy for Biosurveillance, 2012

Model Objective

The specific objective for developing and implementing the model

Objective Categories

- Study disease process
- Evaluate control strategy
- Assess economic impact
- Retrospective analysis
- Contingency planning
- Resource planning
- Training
- Surveillance targeting
- Tactical decision support
- Predict expected number of cases for financial resource planning
- Predict expected case incidence
- What if exercise

Model Scope

Specificity and Granularity

Scope Categories

- Specific outbreak
- Specific disease and location
- Specific disease
- Specific location
- General framework / platform

A model that is highly specified for a particular population, time, or location may not be applicable to other populations, times or places - Garner 2011

The difference between a model and a model platform is in the degree of specificity and granularity - both are still 'models' and referred to as such in the literature

Model: Framework

BSV Goals

BSV goals developed in data streams framework - Margevicius et al., 2014

Biosurveillance Goals

Early warning of health threats

Surveillance that enables the identification of potential threats, including emerging and re-emerging diseases, that may be undefined or unexpected

Early detection of health events

Surveillance that enables identification of disease outbreaks (either natural or intentional in origin), or events that have occurred, before they become significant

Situational awareness

Surveillance that monitors the location, magnitude, and spread of an outbreak or event once it has occurred

Consequence Management

t

Surveillance that assesses impacts and informs response to an outbreak or event

Baseline Awareness

Information that can inform and facilitate the achievement of the above surveillance goals and can be related to population demographics, and health, the natural, social and built environment and underlying disease patterns and characteristics

Model: Framework

"A conceptual model is a verbal or graphical representation of the system under study. Ideally it should be formulated into a document which describes the chosen modeling methods, model assumptions and parameter estimates" - Garner 2011

By using the term Conceptual Model, the overall approach of the model can be described and separated from the details associated with the operational aspects of the model.

Model Types

Risk Mapping

Risk factor analysis displayed spatially or spatio-temporally

Types

- Occurrence Mapping
- Ecological Niche Modeling
- Hotspots, spot maps
- Spatial Risk Mapping
- Spatial-Temporal Risk Mapping
- Neural Net
- Risk Factor Analysis

Anomaly Detection

Alerts over thresholds; finding patterns in data that do not conform to expected behavior

Outbreak detection algorithms; threshold algorithms

Algorithms developed to detect changes from expected results, usually used on surveillance data, can be temporal, spatial, or space-time detection algorithms

Other Anomaly detection algorithms; threshold algorithms

Algorithms developed to detect changes in 'fringe' areas (i.e. transition areas between highly seasonal endemic areas and epidemic areas)

- Regression (GLM)
- Time Series (ARIMA)
- Smoothing
- Hidden Markov
- Wavelet
- EWMA (exponentially weighted moving average)
- MA (moving average)
- CUSUM (cumulative sum)
- MPM (moving percentile method)
- RLS (Recursive Least Squares)
- Coefficient of variation

Specific algorithms (may also be captured under tools)

The term 'fringe' is used to show transition areas from highly seasonal endemic areas to epidemic. Map 'fringes' and look for shifts in 'fringes' to detect areas of high inter-annual variability of transmission (high variability of seasonally or annually averaged incidence) (Morse, SME response)

Model: Framework

Conceptual Model

Disease Dynamics

Progress and /or behavior of disease within a host or population

Disease Model

Epidemiological model describing disease spread /transmission, can be used for both host and vector populations

- States used in model (S,E,I,R, …)
- Heterogeneity
- Resolution
- Structure

Equation-Based

Model built from mathematical equations (differential or difference) that consider disease dynamics at a population or compartmental level

Components
- Stochastic
- Deterministic
- Discrete Time
- Continuous Time
- Linear/nonlinear

Kermack and McKendrik Mass Action aggregate model 1927

Simulation

Model that can simulate disease dynamics down to an individual level

Names of specific Types
- Cellular automata
- Metapopulation
- Individual-based
- Agent-based

Network Structure

The structure used to define the relationships underlying the disease transmission model

Names of specific Types
- Household
- Small -world
- Lattice

Auxillary Models

Non-disease models

Types

- Risk /benefit analysis
- Economic assessment
- Resource planning/budgeting
- Airborne transmission

Possible broader term:

Consequence Assessment- Process of identifying or evaluating the potential or actual effects of an event, incident, or occurrence. Term from DHS Risk Lexicon, 2008, also Forde-Folle 2011

Model: Framework

Conceptual Model

Model Tools

	Type/Name
Computational	▪ Markov Chain Monte Carlo Techniques<br>▪ Monte Carlo<br>▪ GLM (Generalized Linear Model)
Machine Learning<br>Rule-based algorithms	▪ Neural Networks<br>▪ MaxEnt (maximum entropy)
Regression	▪ Linear<br>▪ Logistic<br>▪ Probit<br>▪ ARIMA, SARIMA<br>▪ Box-Jenkins
Statistical	▪ Bayesian<br>▪ Markov chain<br>▪ EWMA (exponentially weighted moving average)<br>▪ MA (moving average)<br>▪ CUSUM (cumulative sum)<br>▪ MPM (moving percentile method)<br>▪ RLS (Recursive Least Squares)

Tool Purpose

- Model Fit
- Model Validation
- Parameter Estimation
- Parameter Optimization
- Time Series Analysis
- Threshold Detection
- Movement between disease states

Model: Framework

Model Inputs

Category	Sub-Categories (not exhaustive)
**Disease**<br>Data relevant to the natural history of the disease; epidemiological information	▪ Reproductive Number<br>▪ Latent, incubation and infectious periods
**Host Population**<br>Data relevant to the infected population	▪ Demographics<br>▪ Density<br>▪ Mobility<br>▪ Behaviour<br>▪ Spatial Distribution<br>▪ Phenotype / Genotype<br>▪ Heterogeneity
**Vector/Reservoir**<br>Data relevant to the vector or reservoir populations	▪ Densities<br>▪ Biting Rates<br>▪ Mobility<br>▪ Behaviour
**Environment**<br>Data relevant to the natural, built and social environment	▪ Climate<br>▪ Temperature<br>▪ Geography<br>▪ Land use<br>▪ Natural Disasters<br>▪ Population displacement<br>▪ Regulations<br>▪ Economics
**Control Efforts**<br>Data relevant to mitigation, interventions and consequence management	▪ Vaccination<br>▪ Isolation<br>▪ Social distancing<br>▪ Culling<br>▪ Biosecurity Measures<br>▪ Pharmaceutical

also
Physical, economic, technological, management, socio-political, social behaviour

Model: Framework

Model Outputs

Identification and description of constraints, caveats and suppositions associated with the developed conceptual model

Output Categories (examples)

- Study disease process
- Evaluate control strategy
- Assess economic impact

Both categorical and detailed model outputs described

Model Assembly

How the overall conceptual model is put together

- Single
- **Multiple**
- **Modular**
- **Hybrid**

Assumptions and Limitations

Identification and description of constraints, caveats and suppositions associated with the developed conceptual model

Regarding

- Disease
- Location
- Model components
- Parameters
-

Assumptions and limitations clearly documented and available for review

Model: Framework

Model Utility

Data

Indicator

- Data sources required
- Availability of data sets
- Data is spatially referenced
- Accuracy and completeness of data
- Documentation

Verification and Validation

Confidence in the model for accurate and credible outputs

- Model verification: conceptual model has been adequately translated into formula or computer code and performs as intended - no coding or logic errors.
- Model validated for purpose built
- Sensitivity analysis of parameters
- Uncertainty analysis (input, output)
- Comparison with other models
- Comparison of model with real system
- Model tested outside of developer team
- Documentation

"The purpose of model evaluation is not to demonstrate that a model is a true or accurate representation of a system, but to subject it to sufficient scrutiny so that it may be used with an appropriate degree of confidence to aid decision-making" - Reeves 2011

Transparency: assumptions clearly documented and available for review

Operations

The ability to use the model in an operational setting

- Model and developer team Accessibility
- Funding Support
- Model used for decision support
- Extensibility
- Model adaptation time
- Source Code / Software availability
- Hardware platform, Operating System, coding language
- Software application needed to visualize results
- Computational time
- Cost to Implement
- Documentation

Extensibility:
How could the model be extended to another disease or location, by 1) only changing parameter values, 2) changing the conceptual model, 3)modifying the code / mathematics 4) software uses hard-coded parameter values vs data files
