# Supplementary material for: The Biosurveillance Analytics Resource Directory (BARD): Facilitating the Use of Epidemiological Models for Infectious Disease Surveillance
Source: PLoS One. 2016 Jan 28;11(1):e0146600. doi: 10.1371/journal.pone.0146600 (PMC4731202; doi:10.1371/journal.pone.0146600)
Supplement: S2 File — (PDF) [file pone.0146600.s002.pdf]

Supplement 2 – BARD usability document

Towards a standardized framework for describing epidemiological models: characterizing models in an operational context

Kristen J Margevicius[1], Nicholas Generous[1], Esteban Abeyta[1], Ben Althouse[2], Howard Burkom[3], Lauren Castro[1], Ashlynn Daughton[1], Sara Y. Del Valle[1], Geoffrey Fairchild[1], James M. Hyman[4], Richard Kiang[5], Andrew P. Morse[6], Carmen M. Pancerella[7], Reid Priedhorsky[1], Laura Pullum[8], Arvind Ramanathan[8], Jeffrey Schlegelmilch[9], Aaron Scott[10], Kirsten J Taylor-McCabe[1], Alessandro Vespignani[11] and Alina Deshpande[1]*

Biosurveillance Analytics Resource Directory (BARD): Usability Requirements

Los Alamos National Laboratory, Los Alamos, NM

1 Introduction **2**

2 Assumptions **2**

3 Use cases **3**

3.1 Primary use cases 3

3.1.1 Model selection 3

3.1.2 Model follow-up 3

3.1.3 Data curation 3

3.2 Secondary use cases 4

3.2.1 Gap analysis 4

3.2.2 New model 4

3.3 Tertiary use cases 4

3.3.1 About 4

3.4 Non use cases 4

3.4.1 Evaluation 4

3.4.2 Modeling 4

4 Personas **5**

4.1 Primary personas 5

4.1.1 Arlene the global health analyst 5

4.1.2 Bret the U.S. agency analyst 5

4.1.3 Don the local public health practitioner 6

4.1.4 Cindy the model developer 6

4.2 Secondary personas 7

4.2.1 Franklin the data curator 7

4.3 Non-personas 7

4.3.1 Emily the decision maker 7

4.3.2 Fiona the reporter 7

Introduction

The Biosurveillance Analytics Resource Directory (BARD) is a catalogue of models of disease outbreaks, defined as unusually high disease incidence at a given time and location. Included models are designed for outbreak prediction (estimating the probability that an outbreak will occur) and/or forecasting (estimating the "extent and locations of disease spread" once an outbreak has occurred).[1]

It contains information about operational models that has been systematically categorized and curated. The BARD is designed to help users rapidly and reliably select epidemiological models appropriate for their real-world situations. In particular, it offers domain-specific search tools several orders of magnitude more efficient in user time and effort than general web tools like Google.

This document describes the usability requirements for the BARD; that is, who will be using the app and what tasks they will be using it for. It does not include information on technical implementation (e.g., whether specific information is contained in the database or pulled on demand from other sources). It also avoids specific design ideas (such as widget descriptions) unless they are necessary to illustrate a requirement.

1 Assumptions

We list here the key assumptions upon which the BARD design is based. There is no particular order.

1. People will use the web app with a standard web browser and relatively large screen, at least 1280×768 at ~100 dpi. Specifically, targeting mobile devices is a future project.
2. Users can read English reasonably well (at the 8th grade reading level or better). This is selected as a level that is readily available among the target users, even those who are not native English speakers.
3. Users are proficient in basic computer use; that is, while they may be unfamiliar with the BARD, they are familiar with their operating system, browser, and related tools.
4. Users have medical or public health training and understand relevant jargon.
5. There will be no user manual for the app. Rather, the app will be self-explanatory, with context-specific help available where needed.

[1] Definitions from: https://www.fbo.gov/index?id=0a03c0b0cd05c9ccebfd50e9b7ff8e98

2 Use cases

This section is a comprehensive listing of BARD use cases. Within each category, no ordering is implied.

2.1 Primary use cases

Our core use cases (i.e., key deliverables). People would have the tasks described below and use the app to support these tasks.

2.1.1 Model selection

Given a specific situation, select from the complete set of plausibly applicable models a small set (say 1–3 models) to explore more deeply. This includes:

- Determine which models are available for specific objectives such as disease, location, and biosurveillance objective (prediction or forecasting). Objectives might include answering questions such as:
  - Should schools be closed, and if so which ones?
  - How much vaccine is necessary? Who should be vaccinated first?
  - What is the economic impact of a given outbreak?
- Quickly understand broad properties of these models, such as operational readiness.
- Understand any model well enough to decide whether to evaluate it more fully. That is, the BARD organizes third-party claims about models to help analysts decide whether to follow up in more detail.
- Compare models to understand their differences.
- Be able to compare selected models and understand differences between models and the user's requirements.
- Understand why some models were excluded (for example, if a plausibly expected model is not in the BARD, understand which inclusion criteria it did not meet).

2.1.2 Model follow-up

Given a specific model, access additional resources regarding that model, doing so as quickly as the model allows (e.g., some models can be downloaded from the web, while others require negotiating access with their developers). These resources might include:

- Contact information for model developers.
- Pointers to the model itself (executables and/or source code).
- Pointers to user documentation.
- Pointers to scientific publications and/or technical documentation.

In other words, this use case supports interaction between analysts/model users and model developers.

2.1.3 Data curation

Management of content stored in the BARD. This includes:

- Adding new models.

- Verifying existing model information.
- Updating information about existing models.
- Removing models which turn out to not meet the inclusion criteria.
- Soliciting verification and updates from others.

2.2 Secondary use cases

Other things we want to be relatively useful. We will spend limited effort on their design but not actively hinder or break them.

2.2.1 Gap analysis

Understand the current state of the art in operational models for a given context (disease, location, etc.). Identify and characterize gaps and challenges in this state of the art.

2.2.2 New model

Understand and characterize a new model.

2.3 Tertiary use cases

Additional things people do that are not so important. These tend to be things that are weakly related to the BARD's purpose, but they are still important to enumerate. We will spend very little design time here.

2.3.1 About

Learn about the database, its team, funding, etc.

2.4 Non use cases

We will not support these; they are currently or permanently out of scope.

2.4.1 Evaluation

The BARD does not include information about third-party evaluation or verification of models. That is, there are no recommendations provided, only information produced by model developers. In particular:

- The maintainers of the catalogue do not perform evaluations.
- The catalogue does not include information on third-party evaluations of the models.

These activities are beyond the scope of the current project.

2.4.2 Modeling

The BARD is not a modeling environment and does not support running models within the tool.

3 Personas

3.1 Primary personas

These are the main personas we will design for. No ordering is implied. **This list should be as short as practicable** (no more than 4); it is better to pick a few representative personas rather than a longer list with relatively minor variations.

3.1.1 Arlene the global health analyst

- **Organization:** National or global health organization; e.g., CDC, WHO, HHS
- **Goals:**
  - Assess the probable future impact of specific diseases in specific locations.
  - In response to an outbreak, forecast its future course in order to make recommendations for response.
  - Communicate this awareness and recommendations to decision makers.
  - Understand the resources that are available with respect to specific possible scenarios. That is, given a particular crisis (real or imagined), understand which tools are available to respond to that crisis.
  - Understand the current status and probable future course of research and development for models of specific diseases, including those that currently lack reliable surveillance and forecasting.
- **Education:** M.S., Ph.D., or M.D. in epidemiology, public health, or medicine
- **Behavior/attitude:** May be wary of the use of models for decision-making; more likely relies on multiple criteria and will view models as supplementary.
- **Tech savviness:** Moderate. Uses computers throughout her work, but is not interested in technology for its own sake.
- **Knowledge of disease models:** Rudimentary; limitations and assumptions associated with model output will not be readily apparent.

3.1.2 Bret the U.S. agency analyst

- **Organization:** DOD, NCMI, DHS, etc.
- **Goals:**
  - Assess biological and health threats to the general U.S. population as well as armed forces in the U.S. and abroad.
  - Forecast future threats to U.S. interests.
  - Efficiently communicate these assessments and forecasts to decision makers.
  - Integrate classified and unclassified information while carefully following security requirements protecting the former.

- **Education:** Varied. Highly proficient in area of expertise, this may be a class of diseases, a class of threat scenarios, geographic area, etc.
- **Behavior/attitude:** Feels constantly "under the gun" to provide reliable information as quickly as possible. Most interested in readily available models that provide immediate and actionable results. Sometimes willing to collaborate with model developers to build new models that meet long-term needs.

- **Tech savviness:** Moderate to high.
- **Knowledge of models:** Moderate. Frequently uses models in decision-making but does not have the time to understand the finer details of each model being used.
- ***Note:*** *While similar to Arlene, Bret has a singular focus on threats to U.S. interests. Also, he has access to the broader set of tools in the BSV Ecosystem.*

3.1.3 Don the local public health practitioner

- **Organization:** City, county, or state level public health agency
- **Goals:**
    - Build and maintain situational awareness regarding any diseases which are causing current or potential local problems.
    - Make decisions about local strategy and specific local interventions.
    - Explain and justify these decisions to local politicians and the public.
- **Education:** B.S. or M.S. in public health.
- **Behavior/attitude:** Focused on local problems and local solutions; less interested in the nationwide or global picture. Overworked and under-budgeted. Committed to his responsibility, but his department has suffered from multiple rounds of budget cuts and is understaffed. Wants to quickly complete his task and move on to the next thing. Lacks budget to modify models or perform complex parameterization; i.e., he needs something already tested that is ready to go.
- **Tech savviness:** Moderate. Uses computers throughout his work, but is not interested in technology for its own sake.
- **Knowledge of models:** Varied. Will use models to help in decision-making., but this is not a primary interest or expertise. Limitations and assumptions in model output may not be readily apparent.

3.1.4 Cindy the model developer

- **Organization:** Academic institution (university, national lab, industrial research)
- **Goals:**
    - Create the "best" model for a given scenario.
    - Understand competing models in his or her area of interest.
    - Publish in the peer-reviewed literature.
    - Be judged well by funding agencies and academic peers.
    - Use model in research leading to publication in the peer-reviewed literature.
    - Tackle the most interesting problems in the most innovative way.
    - Publicize her work.
- **Education:** M.S. or Ph.D. in bioinformatics, computer science, epidemiology, public health, statistics, or mathematics.
- **Behavior/attitude:** Detail oriented and competitive. Can be protective of her team's models, e.g., may not want to share information if she thinks it might be depicted or evaluated negatively, or that by giving it away she might lose a competitive advantage. Continually worries about funding and related issues like tenure. Focused more on model innovation than their practical use.
- **Tech savviness:** Moderate to high. May be well versed in the mathematics and theory of modeling while having a weaker understanding of its practical computational aspects.
- **Knowledge of models:** Exceptionally high in her own research specialty; moderate for models outside her area of interest.

3.2 Secondary personas

We will consider these personas in our design, but they are not our focus and will not be considered in usability testing.

3.2.1 Franklin the data curator

- **Organization:** FFRDC, private contractor, university
- **Goals:**
  - Add new content on request of sponsors.
  - Keep existing BARD content correct and up to date.
  - Promote the value of well-maintained data.
- **Education:** B.S., M.S., or Ph.D. in public health, epidemiology, or a related field.
- **Behavior/attitude:** Busy with other things. Concerned about maintaining resources (time, funding, personnel) for ongoing data curation vs. new features and projects.
- **Tech savviness:** Moderate. More concerned with information quality than the technical means of accomplishing this.
- **Knowledge of models:** Moderate. Broad exposure to a variety of different models, but not deeply immersed in the technical, mathematical, or computational details.

3.3 Non-personas

Design for these personas is explicitly excluded.

3.3.1 Emily the decision maker

Decision makers are indirect users of the BARD. They will rely on reports, presentations, etc. prepared by the direct users noted above.

3.3.2 Fiona the reporter

News people will rely on the above personas for a more digestible view of what is going on.
